# The Postoperative Visual and Refractive Outcomes of Trifocal and Extended Depth-of-Focus Intraocular Lenses in Patients with Different Biometric Characteristics

**DOI:** 10.3390/diagnostics14161717

**Published:** 2024-08-07

**Authors:** Chia-Yi Lee, Hung-Chi Chen, Ie-Bin Lian, Jing-Yang Huang, Shun-Fa Yang, Chao-Kai Chang

**Affiliations:** 1 Institute of Medicine, Chung Shan Medical University, Taichung 40201, Taiwan; 2 Nobel Eye Institute, Taipei 10041, Taiwan; 3 Department of Ophthalmology, Jen-Ai Hospital Dali Branch, Taichung 41265, Taiwan; 4 Department of Ophthalmology, Chang Gung Memorial Hospital, Linkou, Taoyuan 33305, Taiwan; 5 Center for Tissue Engineering, Chang Gung Memorial Hospital, Linkou, Taoyuan 33305, Taiwan; 6 Department of Medicine, Chang Gung University College of Medicine, Taoyuan 33305, Taiwan; 7 Institute of Statistical and Information Science, National Changhua University of Education, Chunghua 50007, Taiwan; 8 Department of Medical Research, Chung Shan Medical University Hospital, Taichung 40201, Taiwan; 9 Department of Optometry, Da-Yeh University, Chunghua 51591, Taiwan

**Keywords:** extend depth-of-focus, trifocal, uncorrected distance visual acuity, spherical equivalent, axial length

## Abstract

We aimed to survey the potential correlation between biometric parameters and postoperative outcomes after implanting extended depth-of-focus (EDOF) intraocular lenses (IOLs) and trifocal IOLs. A retrospective cohort study was conducted, and patients receiving EDOF or trifocal IOL implantations were included. In total, 36 and 26 eyes were enrolled in the EDOF and trifocal groups, respectively. The primary outcomes of this study were the postoperative uncorrected distance visual acuity (UDVA), uncorrected near visual acuity (UNVA), and spherical equivalent (SE). The generalized linear model was applied to evaluate the adjusted odds ratio (aOR) and 95% confidence intervals (CIs) of primary outcomes in patients with different biometric characters. The final UDVA of the EDOF group was significantly better than that of the trifocal group (*p* = 0.020), and the UNVA and SE did not show significant differences between the two groups throughout the postoperative period (all *p* > 0.05). In a multivariable analysis, the UDVA was significantly better in the EDOF group than in the trifocal group (*p* = 0.038). For the subgroup analysis, the high axial length (AXL) value correlated to a lower postoperative UDVA in the EDOF group (both *p* < 0.05). Additionally, a large white-to-white (WTW) diameter was related to worse postoperative UNVA in the trifocal group (*p* = 0.042), and a high AXL was associated with higher SE in both the EDOF and trifocal groups (both *p* < 0.05). In conclusion, a high AXL correlates to worse postoperative outcomes in both the EDOF and trifocal IOLs, and trifocal IOL outcomes could be affected by large WTW diameters.

## 1. Introduction

The cataract is a prevalent ophthalmic disorder contributing to blindness in more than 90 million people worldwide [1]. The common symptoms of cataract formation include progressively blurry vision, hale, photophobia, difficulty seeing at night, and monocular diplopia [1,2]. Currently, the only intervention to resolve the reduced vision caused by cataracts is surgery [3]. In general, visual acuity after cataract surgery is significantly improved [4,5], and multiple types of intraocular lenses (IOLs) can be chosen to meet the requirements for distance and near vision [6,7].

Presbyopia-correcting IOLs, including trifocal and extended depth-of-focus (EDOF) lenses, were introduced in 2000 and are used in several countries [8,9]. In a previous study, the overall mean postoperative uncorrected distance visual acuity (UDVA) after the presbyopia-correcting IOL implantation was approximately 0.10 LogMAR [10]. Additionally, the postoperative refractive status was acceptable in both EDOF and trifocal IOLs, with a high percentage of patients reaching the acceptable postoperative SE in both types of IOLs [11,12]. Recently, a new type of diffractive trifocal IOL showed good distance and near visual acuity, and subjective satisfaction was also acceptable [13,14]. In addition, a newly introduced EDOF IOL demonstrated a wider range of vision and equal distance visual acuity compared to monofocal IOLs [15], and patient satisfaction one year after the cataract surgery has been reported as satisfactory [16]. However, certain postoperative symptoms, including low visual acuity, glare, hale, and diplopia, were found in patients who received presbyopia-correcting IOL implantation [17,18]. In severe cases, IOL extraction and replacement could be warranted to resolve the persistent impaired visual quality [19].

Previous studies have proposed some predisposing factors to predict worse postoperative outcomes of presbyopia-correcting IOLs [20,21]. In a study that examined the initial form of trifocal IOL [21], a strong correlation was found between the large angle alpha and the poorer postoperative UDVA. In addition, another study revealed that a large lens tilt is associated with lower postoperative near vision in presbyopia-correcting IOLs [20]. Regarding the biometric parameters, a long axial length (AXL) reduces the postoperative visual acuity after cataract surgery with monofocal IOL implantation [22]. In addition, the anterior chamber depth (ACD) would influence the outcome of general cataract surgery in microphthalmos, which usually has worse surgical outcomes [23]. However, a scant study investigated the effect of preoperative biometric indexes on the postoperative outcomes of patients who received trifocal and EDOF IOL implantation. Because the biometric index, including the AXL, ACD, and corneal diameter, could alter the postoperative outcomes in monofocal IOL implantation [22,23], some of them may also affect the visual and refractive outcomes of presbyopia-correcting IOLs.

Consequently, this study aims to evaluate the potential correlation between preoperative biometric parameters and postoperative outcomes in patients who received EDOF and trifocal IOL implantation. The patients with different biometric characters were separated and analyzed in the statistical analysis. We expect that a long AXL will contribute to worse visual and refractive outcomes in both types of IOLs, while the roles of ACD and corneal diameter on postoperative outcomes are relatively harder to predict.

## 2. Materials and Methods

### 2.1. Subject Selection

A retrospective cohort study was performed at the Nobel Eye Institute, which has more than 12 branches of clinics in Taiwan. Individuals selected for this study were (1) aged between 45 and 100 years, (2) recipients of a complicated cataract or senile cataract diagnosis, (3) people who had cataract surgery and EDOF or trifocal IOL implantation at the Nobel Eye Institute, and (4) people who followed up at the Nobel Eye Institute for more than one month. Moreover, the following exclusion criteria were utilized to exclude individuals with specific statuses: (1) their corrected distance visual acuity (CDVA) before surgery was worse than hand motion, (2) the presence of total corneal opacity or previous central-involved keratitis, (3) the presence of prominent retinal diseases such as rhegmatogenous retinal detachment or proliferative diabetic retinopathy, (4) the presence of neovascular glaucoma, (5) the presence of optic neuropathy, (6) the presence of previous eyeball rupture episodes, and (7) the receipt of monovision (planned residual myopia) refractive management. Then, the participants were divided into the EDOF group and the trifocal group according to their IOL types. In addition, this study only considered the first eye of each participant. After the whole process, a total of 62 eyes from 62 patients were enrolled, and 36 and 26 eyes were categorized into the EDOF and trifocal groups, respectively.

### 2.2. Surgical Techniques

All the cataract surgeries in this study were accomplished by one experienced cataract specialist (C.-Y.L.) and one phacoemulsification device (Quatera, Carl Zeiss, Göschwitzer Str., Jena, Germany). The main incision was made using a superior approach, and the ophthalmic viscoelastic device was injected from the main incision. After accomplishing the continuous curvilinear capsulorhexis, hydrodissection was performed before side-port creation. The phaco chop technique was used to clear the nucleus, and the residual cortex was extracted with an infusion–aspiration probe. One type of EDOF IOL (AT LARA^®^, Carl Zeiss, Göschwitzer Str., Jena, Germany) and one type of trifocal IOL (AT LISA^®^ tri, Carl Zeiss, Göschwitzer Str., Jena, Germany) were implanted into the bag, and the residual ophthalmic viscoelastic device was cleaned with an infusion–aspiration probe. A hydroseal technique was adopted to seal the main incision and side port, and then, tobradex ointment was applied. After the cataract surgery, levofloxacin eyedrops, prednisolone eyedrops, and the tobradex ointment were instilled for 7 days; then, the medications were changed to dexamethasone/neomycin eyedrops for another 7 days. After that, sulfamethoxazole and fluorometholone eyedrops were instilled for about three weeks.

### 2.3. Ocular Examination

All patients who received cataract surgery underwent standardized operation examinations at any clinic of the Nobel Eye Institute. The preoperative exams included the following tests: manifest refraction with UDVA and CDVA; cycloplegic refraction of the sphere and cylinder power using an autorefractor (KR-8900, Topcon, Itabashi-ku, Tokyo, Japan); the measurement of intraocular pressure (IOP) using a pneumatic tonometer (NT-530, Nidek Co. Ltd., Gamagori, Japan); an assessment of central corneal thickness (CCT); measurement of steep keratometry (K) and flat K; the evaluation of corneal astigmatism; the determination of the kappa angle; the measurement of the scopic pupil diameter; the calculation of the corneal eccentricity index (CEI); the assessment of higher order aberrations (HOA) and spherical aberration (SA) using a topographic machine (TMS-5, Tomey Corporation, Nishi-Ku, Nagoya, Japan); and the measurement of the AXL, ACD, lens thickness (LT), and corneal diameter presented as white-to-white (WTW) using a biometry machine (IOL Master 700, Carl Zeiss, Göschwitzer Str., Jena, Germany). The endothelial cell density (ECD), coefficient of variant (CV), and hexagonality (HEX) were measured using a specular microscope (CEM-530, Nidek Co. Ltd., Gamagori, Japan). The postoperative exams included UDVA, uncorrected near visual acuity (UNVA), IOP, manifest sphere, and cylinder powers. The postoperative exams were conducted using the same devices the preoperative exams used. The information was obtained before the surgery, one week postoperatively, two weeks postoperatively, and one month postoperatively. In this study, an IOL calculator (version 2.0) provided by ASCRS was utilized for all the IOL calculations, and SE was defined as the sum of the sphere power and half of the cylinder power.

### 2.4. Statistical Analysis

SPSS version 20.0 (SPSS Inc., Chicago, IL, USA) was administered for statistical analysis in this study. The Shapiro–Wilk test was administered to investigate the normality of the study population, which revealed no normal distribution (*p* < 0.05). The descriptive analysis was administered to exhibit age, sex, pre-existing ocular disorders, UDVA, CDVA, myopia degree, astigmatism degree, topographic parameters, endothelial indexes, and biometric parameters. Then, the Chi-square test and Mann–Whitney U test were administered to compare the preoperative and postoperative features between the EDOF and trifocal groups. Then, the generalized linear model was administered to evaluate the UDVA, UNVA, and SE (as absolute value) three months postoperatively between the two groups. The generalized linear model yielded the adjusted odds ratio (aOR) with a 95% confidence interval (CI) between the two groups after adjusting age, sex, and preoperative SE. For the postoperative visual acuity and refraction in patients with different biometric conditions, the study population was separated into those with high AXL (longer than 26 mm), short ACD (lower than 3 mm), and large WTW (>12 mm). In the next step, the generalized linear model was administered again to evaluate the postoperative UDVA, UNVA, and SE (as absolute values) in different subgroups. A *p*-value < 0.05 was defined as statistically significant, and a *p*-value lower than 0.001 was demonstrated as *p* < 0.001.

## 3. Results

The initial characteristics of the two groups are demonstrated in Table 1. The mean age was 56.17 ± 8.80 in the EDOF group and 62.71 ± 7.59 in the trifocal group, respectively. The difference in mean age did not illustrate a significant difference between the two groups (*p* = 0.061). In addition, the sex distributions and the ratio of systemic diseases were statistically insignificant between the groups (*p* > 0.05). In terms of the preoperative parameters, the mean UDVA and CDVA were statistically identical between the two groups (both *p* > 0.05), and the preoperative SE, as well as other preoperative parameters, also showed similar values between the two groups (all *p* > 0.05) (Table 1).

**Figure 1:**
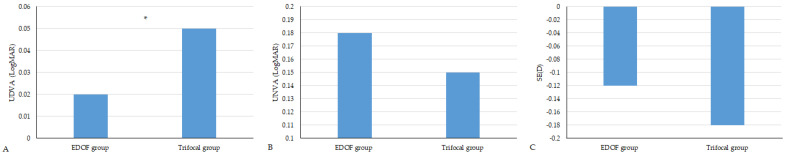
The final postoperative outcomes between the two groups. (**A**) The final uncorrected visual acuity between groups; (**B**) the final uncorrected near visual acuity between groups; and (**C**) the final spherical equivalent between groups. D: diopter; EDOF: extended depth-of-focus; UDVA: uncorrected distance visual acuity; UNVA: uncorrected near visual acuity; SE: spherical equivalent. * denotes significant differences between groups.

One day after cataract surgery, the UDVA was 0.05 ± 0.12 in the EDOF group, similar to that in the trifocal group (0.09 ± 0.09) (*p* = 0.159). The final UDVA of the EDOF group was significantly better than that in the trifocal group (*p* = 0.020) (Table 2). On the other hand, there was not a significant difference in UNVA between the two groups throughout the postoperative period (all *p* > 0.05) (Table 2), and the postoperative SE also displayed similar values between the EDOF and trifocal groups (both *p* < 0.05) (Table 2). Concerning the final postoperative UDVA, UNVA, and SE between the two groups after adjustment, UDVA was significantly better in the EDOF group than the trifocal group (aOR: 1.162, 95% CI: 1.007–1.297, *p* = 0.038) (Table 3), and both UNVA and SE were statistically identical between the EDOF and trifocal groups (both *p* > 0.05) (Table 3). The values for the final postoperative UDVA, UNVA, and SE between the two groups are presented in Figure 1.

In the subgroup analysis stratified by different biometric parameters, the high AXL value correlated to lower postoperative UDVA in both the EDOF group (aOR: 1.429, 95% CI: 1.221–1.657, *p* = 0.005) and the trifocal group (aOR: 1.394, 95% CI: 1.198–1.596, *p* = 0.011) (Table 4). Additionally, the UNVA was not affected by any of the biometric parameters in the EDOF group (all *p* > 0.05), but a large WTW diameter related to worse postoperative UNVA in the trifocal group (aOR: 1.349, 95% CI: 1.008–1.527, *p* = 0.042) (Table 5). On the other hand, a high AXL was associated with higher SE in both the EDOF (aOR: 1.231, 95% CI: 1.019–1.348, *p* = 0.031) and trifocal (aOR: 1.326, 95% CI: 1.195–1.676, *p* = 0.019) groups, and the short ACD was associated with a marginally higher postoperative SE in the trifocal group (aOR: 1.261, 95% CI: 0.999–1.573, *p* = 0.050) (Table 6).

## 4. Discussion

In this study, the postoperative UDVA was better in the EDOF group than the trifocal group after adjusting several preoperative factors. Moreover, the higher AXL was associated with worse visual and refractive outcomes in both the EDOF and trifocal groups. In addition, the large WTW diameter would alter the refractive outcomes in the trifocal group.

The high AXL correlated to the worse postoperative UDVA and higher residual SE in both the EDOF and trifocal groups. In previous studies, the long AXL contributed to lower postoperative visual acuity using monofocal IOLs [22], and the overall UDVA in patients with high myopia or long AXL was lower [24]. In a previous study that discussed the factors that influence the results of trifocal IOL implantation, the large angle alpha was also related to lower postoperative visual acuity [21]. In addition, the high preoperative myopia and large angle kappa were associated with lower postoperative satisfaction [25,26]. A study evaluated the predisposing factors contributing to worse postoperative outcomes in the latest generation of EDOF and trifocal IOLs. To our knowledge, this study may be preliminary research demonstrating the potential risk factor of the EDOF and trifocal IOLs. Additionally, all the cataract surgeries were performed by one surgeon; thus, the surgical protocols and techniques among all the patients were identical. In addition, the preoperative status, including the age, sex, and refractive status, were adjusted in the multivariable analysis, which can reduce the influence of these confounding factors. As a consequence, the high AXL may be an independent risk factor for worse postoperative outcomes in patients receiving EDOF and trifocal IOL implantation. A possible explanation for the correlation between high AXL and worse postoperative visual and refractive outcomes in both groups is that a long eyeball contributes to a large capsular bag and a higher chance of IOL rotation after cataract surgery [27,28,29]. Consequently, the stability of IOL placement is relatively lower in the high AXL population, which contributes to IOL movement, which causes higher postoperative refractive error and worse postoperative visual acuity [30]. Additionally, the high AXL will decrease the accuracy of IOL calculation; thus, the postoperative SE could be higher in those with high AXL [31]. We speculate that the inaccuracy of IOL calculation contributes more to the correlation between high AXL and lower postoperative UDVA in this study. Because a large proportion of our patients received non-toric IOL implantation, the effect of IOL rotation due to a large capsular bag on postoperative UDVA could be minimal. The large capsular bag might lead to some IOL tilt or subluxation, influencing the postoperative UDVA, but the degrees may not be prominent. Still, the mean postoperative UDVA and SE in the high AXL group was 0.10 and −0.24 D in the EDOF group and 0.13 and −0.32 D in the trifocal group, which may be acceptable in clinical aspect.

Regarding the influence of other biometric parameters on the postoperative outcomes of trifocal IOL implantation, the short ACD was associated with marginally higher postoperative SE in the trifocal group. The *p*-value of postoperative SE between the short and large ACD subgroups in the trifocal population did not reach a significant difference (*p* = 0.050), and the difference in SE between the two groups was approximately −0.15 D, which did not have a prominent influence in clinical practice. In a previous study, the ACD significantly influenced the judgment of IOL power, in which a 1.5 D error occurred with an ACD change of 1 mm [32]. We speculate that, because the mean ACD in this study was near the mean ACD in the general population, the width of ACD could only contribute to a marginal effect on the postoperative SE in the trifocal group. Conversely, the large WTW diameter related to worse postoperative SE in the trifocal group but not the EDOF group. There was scant research to demonstrate this phenomenon. The large WTW was associated with large pupil diameter in a previous study [33], and the large pupil size could contribute to more visual symptoms and thus may reduce postoperative visual acuity [34,35]. The trifocal group, which was theoretically easier to interfere with by aberrations [21], may experience more dysphotopsia symptoms than the EDOF population; thus, the UNVA was reduced. Another possible mechanism for the worse UNVA in the trifocal group with a large WTW diameter is that the large WTW diameter was also correlated to lens diameter and possible a large capsular bag [36], which can influence the position of trifocal IOLs and contribute to subsequent refractive error formation. Consequently, the fluctuation of IOLs in patients with large capsular bags may cause a reduction in UNVA, especially in the patients who received trifocal IOL implantation. Still, the overall UNVA was numerically better in the trifocal group than the EDOF group in this study, which corresponded to previous experience that EDOF IOLs have a better intermediate vision [14]. In contrast, the trifocal IOLs have a better near vision [14].

Concerning the refraction accuracy in this study, the mean postoperative SE was −0.12 D in the EDOF group, which did not exceed the minimal unit of refraction (−0.25 D) and could indicate the fair predictability of IOL calculation in this study. If we divided the SE into the sphere and cylinder powers, the mean postoperative sphere and cylinder powers were −0.06 D and −0.13 D in the EDOF group. In a previous study that used toric IOLs, the postoperative SE was −0.48 D, and the EDOF group results were comparable to previous experience [37]. On the other side, the postoperative SE of the trifocal group was −0.18 D, which was slightly higher than the −0.12 D in the EDOF group but still an acceptable value compared to the previous study [12]. Regarding the sphere and cylinder power in the trifocal group, the higher SE mainly resulted from the residual sphere power (−0.13 D) but not the residual cylinder power (−0.10 D). The attempted SE in the EDOF and trifocal group was −0.00 D in this study, and a larger difference between targeted and actual SE in the trifocal group than in the EDOF group could imply the influence of biometric parameters. The postoperative cylinder powers in the trifocal group were similar to that in the EDOF group and in the previous research that applied the multifocal toric IOL [38]. In addition, 13 and 8 eyes received toric IOL implantation in the EDOF and trifocal groups, respectively, due to the high preoperative corneal astigmatism. Regarding the refractive results, the difference in postoperative cylinder power between the toric and non-toric IOL implantation was −0.07 D and −0.06 D in the EDOF and trifocal groups, respectively, which were insignificant in the statistical and clinical aspects. Consequently, the high preoperative corneal astigmatism did not relate to significant postoperative astigmatism in this study, and the high preoperative corneal astigmatism may have little contribution to the selection between EDOF and trifocal IOLs. The prominent postoperative astigmatism after cataract surgery may need surgical management like laser vision correction or IOL exchange to resolve the poor vision and possible dysphotopsia [39]. Still, no postoperative management for residual astigmatism is warranted since no prominent postoperative astigmatism or dysphotopsia was noted in this study.

For the visual outcomes of cataract surgery in this study, the mean postoperative UDVA in our study population was 0.02 and 0.05 in the EDOF and trifocal groups. In the earlier study, the mean UDVA was approximately 0.05 to 0.13 in presbyopia-correcting IOLs, and the visual outcomes of this study were compatible with the earlier publication [10]. Concerning the postoperative UNVA, the mean UNVA was 0.18 in the EDOF group. The mean UNVAs in the earlier research used EDOF and trifocal IOLs ranging from 0.24 to 0.06 [40]. In addition, the mean UNVA was 0.15 in the trifocal group, comparable to the mean UNVA in a previous study that applied the trifocal IOLs [41]. Regarding the postoperative visual quality, six patients reported dysphotopsia symptoms in this study, including hale and glare, while no IOL exchange was scheduled to resolve this dysphotopsia, which subsided about two to three months postoperatively. The incidence of dysphotopsia was not inferior to the 10 percent of prominent dysphotopsia symptoms in the previous study using presbyopia-correcting IOLs [42]. Consequently, the surgical quality of this study may be adequate compared to the other research with presbyopia-correcting IOL implantation.

There were a few limitations in this study. Firstly, the retrospective nature of the study design diminished the homogeneity of the study population compared to a prospective one, although the preoperative parameters showed no difference between the two groups. Additionally, the case numbers of the study population were relatively insufficient, as only 62 eyes were enrolled, which could influence the statistical power. In addition, this study did not measure some parameters, such as the posterior corneal curvature and the higher-order aberrations, due to the retrospective design; thus, these prominent confounders for postoperative outcomes cannot be evaluated. Finally, we enrolled all the eyes with non-toric and toric IOL implantations as a single group because the total eye number was not adequate enough to separate the eyes with different preoperative astigmatisms into different subgroups, which would reduce the accuracy of our analysis. Due to the above limitations, the integrity of our results is inferior to a study with prospective design and adequate case numbers; thus, the role of AXL, ACD, WTW, and the toric IOL application on the postoperative outcomes of EDOF and trifocal IOLs cannot be fully confirmed.

## 5. Conclusions

In conclusion, the high AXL correlates to worse postoperative visual and refractive outcomes in the eye receiving trifocal IOLs than in the eyes receiving EDOF IOL. Furthermore, the visual outcome of eye-received trifocal IOL implantation was affected by WTW diameter more easily. Consequently, the EDOF IOL might be considered for the patient’s request for spectacle independence prior to trifocal IOL if the preoperative examination illustrated extreme values of AXL and WTW. If the AXL of the patient was longer than 26 mm, EDOF and trifocal IOLs should not be recommended first. On the other hand, if an individual has an AXL shorter than 26 mm but an ACD lower than 3 mm or a WTW diameter larger than 12 mm, EDOF IOLs should be recommended before trifocal IOLs. Further large-scale prospective studies to investigate whether topographic parameters would influence the outcomes of presbyopia-correcting IOLs, examine the long-term stability of presbyopia-correcting IOLs in individuals with elevated AXL and WTW values, and evaluate the influence of posterior higher-order corneal aberrations on visual and refractive outcomes of presbyopia-correcting IOLs are essential.

## Figures and Tables

**Table 1:** Baseline features in the study populations.

Feature	EDOF Group (*N*: 36)	Trifocal Group (*N*: 26)	*p*
Age (year, mean ± SD)	56.17 ± 8.80	62.71 ± 7.59	0.061
Sex (male:female)	6:30	9:17	0.343
Laterality (right:left)	24:12	13:13	0.179
Systemic disease			0.154
Hypertension	4	2	
Diabetes mellitus	1	0	
Other	1	0	
Refractive surgery	0	1	0.538
UDVA (LogMAR)	0.52 ± 0.28	0.47 ± 0.19	0.441
CDVA (LogMAR)	0.37 ± 0.06	0.41 ± 0.09	0.064
IOP	15.77 ± 2.58	17.11 ± 4.49	0.836
Cycloplegia refraction (D)			
Sphere	−1.79 ± 3.27	−1.82 ± 3.74	0.927
Cylinder	−1.12 ± 0.95	−1.68 ± 0.73	0.073
SE	−2.35 ± 3.39	−2.66 ± 3.84	0.705
Topography			
Steep K	44.08 ± 0.87	42.56 ± 2.03	0.073
Flat K	43.34 ± 0.87	41.64 ± 2.02	0.074
Cylinder power	0.74 ± 0.33	0.92 ± 0.39	0.534
CCT	525.67 ± 36.64	527.57 ± 26.44	0.945
Angle Kappa	0.16 ± 0.12	0.20 ± 0.10	0.534
Pupil diameter	4.05 ± 1.46	3.88 ± 0.45	0.538
CEI	0.58 ± 0.09	0.24 ± 0.38	0.001
HOA	0.25 ± 0.23	0.20 ± 0.09	0.731
SA	0.24 ± 0.10	0.10 ± 0.33	0.534
AXL	26.51 ± 2.19	25.76 ± 2.19	0.474
ACD	3.25 ± 0.45	3.27 ± 0.61	0.836
WTW	11.83 ± 0.23	12.29 ± 0.57	0.234
LT	4.16 ± 0.22	4.49 ± 0.71	0.628
ECD	2917.00 ± 156.03	2656.29 ± 265.90	0.101
CV	32.33 ± 6.02	30.02 ± 2.94	0.445
HEX	59.17 ± 6.82	67.43 ± 7.09	0.053
Femtosecond Laser	6	7	0.103

ACD: anterior chamber depth; AXL: axial length; CCT: central corneal thickness; CDVA: corrected distance visual acuity; CEI: corneal eccentricity index; CV: coefficient of variance; D: diopter; EDOF: extended depth-of-focus; HEX: hexagonality; HOA: higher-order aberrations; IOL: intraocular lens; IOP: intraocular pressure; K: keratometry; LT: lens thickness; *N*: number; SA: spherical aberration; SD: standard deviation; UDVA: uncorrected distance visual acuity; WTW: white-to-white.

**Table 2:** Postoperative visual and refractive outcomes between the two groups.

Outcome	EDOF Group (*N*: 36)	Trifocal Group (*N*: 26)	*p*
UDVA (mean ± SD)			
1 day	0.05 ± 0.12	0.09 ± 0.09	0.159
1 week	0.03 ± 0.06	0.05 ± 0.07	0.258
2 weeks	0.03 ± 0.05	0.06 ± 0.07	0.053
1 month	0.02 ± 0.04	0.05 ± 0.06	0.020 *
UNVA (mean ± SD)			
1 day	0.31 ± 0.29	0.28 ± 0.30	0.674
1 week	0.27 ± 0.26	0.24 ± 0.23	0.657
2 weeks	0.20 ± 0.26	0.17 ± 0.25	0.698
1 month	0.18 ± 0.27	0.15 ± 0.22	0.661
SE (mean ± SD)			
1 day	−0.25 ± 0.20	−0.21 ± 0.23	0.478
1 week	−0.19 ± 0.23	−0.22 ± 0.25	0.664
2 weeks	−0.18 ± 0.27	−0.20 ± 0.30	0.815
1 month	−0.12 ± 0.23	−0.18 ± 0.24	0.304

EDOF: extended depth-of-focus; UDVA: uncorrected distance visual acuity; UNVA: uncorrected near visual acuity; *N*: number; SD: standard deviation; SE: spherical equivalent. * Denotes significant differences between groups.

**Table 3:** The difference in visual and refractive outcomes between the two groups in multivariable analysis.

Outcome	EDOF Group (*N*: 36)	Trifocal Group (*N*: 26)	*p*
UDVA			
Crude OR (95% CI)	Reference	1.231 (1.019–1.348)	0.026 *
aOR (95% CI)	Reference	1.162 (1.007–1.297)	0.038 *
NVA			
Crude OR (95% CI)	Reference	0.924 (0.768–1.285)	0.597
aOR (95% CI)	Reference	0.943 (0.829–1.267)	0.659
SE			
Crude OR (95% CI)	Reference	1.194 (0.907–1.475)	0.385
aOR (95% CI)	Reference	1.132 (0.926–1.388)	0.426

aOR: adjusted odds ratio; CI: confidence interval; EDOF: extended depth-of-focus; *N*: number; UDVA: uncorrected distance visual acuity; UNVA: uncorrected near visual acuity. * Denotes significant differences between groups.

**Table 4:** The correlation between biometric parameters and the postoperative uncorrected distance visual acuity in the two groups.

Subgroup	aOR	95% CI	*p*
High AXL (reference: non-high AXL)			
EDOF group	1.429	1.221–1.657	0.005 *
Trifocal group	1.394	1.198–1.596	0.011 *
Short ACD (reference: non-short ACD)			
EDOF group	1.015	0.884–1.295	0.295
Trifocal group	1.124	0.863–1.274	0.221
Large WTW (reference: non-large WTW)			
EDOF group	0.976	0.749–1.423	0.777
Trifocal group	0.994	0.813–1.359	0.825

ACD: anterior chamber depth; aOR: adjusted odds ratio; AXL: axial length; CI: confidence interval; EDOF: extended depth-of-focus; WTW: white-to-white. * denotes a significant correlation to postoperative uncorrected distance visual acuity.

**Table 5:** The correlation between biometric parameters and the postoperative uncorrected near visual acuity in the two groups.

Subgroup	aOR	95% CI	*p*
High AXL (reference: non-high AXL)			
EDOF group	1.112	0.921–1.468	0.334
Trifocal group	1.264	0.977–1.601	0.106
Short ACD (reference: non-short ACD)			
EDOF group	0.968	0.823–1.254	0.276
Trifocal group	1.083	0.728–1.288	0.309
Large WTW (reference: non-large WTW)			
EDOF group	1.236	0.961–1.618	0.112
Trifocal group	1.349	1.008–1.527	0.042 *

ACD: anterior chamber depth; aOR: adjusted odds ratio; AXL: axial length; CI: confidence interval; EDOF: extended depth-of-focus; WTW: white-to-white. * denotes a significant correlation to uncorrected near visual acuity.

**Table 6:** The correlation between biometric parameters and the postoperative spherical equivalent in the two groups.

Subgroup	aOR	95% CI	*p*
High AXL (reference: non-high AXL)			
EDOF group	1.231	1.019–1.348	0.031 *
Trifocal group	1.326	1.195–1.676	0.019 *
Short ACD (reference: non-short ACD)			
EDOF group	1.148	0.962–1.403	0.139
Trifocal group	1.261	0.999–1.573	0.050
Large WTW (reference: non-large WTW)			
EDOF group	0.912	0.811–1.316	0.559
Trifocal group	0.987	0.870–1.229	0.698

ACD: anterior chamber depth; aOR: adjusted odds ratio; AXL: axial length; CI: confidence interval; EDOF: extended depth-of-focus; WTW: white-to-white. * denotes a significant correlation to the spherical equivalent.

## Data Availability

The data used in this study are available from the corresponding authors upon reasonable request.
